# U.K. Intensivists’ Preferences for Patient Admission to ICU: Evidence From a Choice Experiment

**DOI:** 10.1097/CCM.0000000000003903

**Published:** 2019-10-11

**Authors:** Christopher R. Bassford, Nicolas Krucien, Mandy Ryan, Frances E. Griffiths, Mia Svantesson, Zoe Fritz, Gavin D. Perkins, Sarah Quinton, Anne-Marie Slowther

**Affiliations:** 1 Warwick Medical School, University of Warwick, Coventry, United Kingdom.; 2 University Hospitals Coventry and Warwickshire NHS Trust, Coventry, United Kingdom.; 3 Health Economics Research Unit, Institute of Applied Health Sciences, University of Aberdeen, Aberdeen, United Kingdom.; 4 University Health Care Research Center, Faculty of Medicine and Health, Örebro University, Örebro, Sweden.; 5 The Healthcare Improvement Studies (THIS) Institute, Cambridge University, Cambridge, United Kingdom.; 6 University Hospitals Birmingham NHS Foundation Trust, Heartlands Hospital, Birmingham, United Kingdom.

**Keywords:** choice experiment, decision-making, intensive care admissions, intensive care triage

## Abstract

Deciding whether to admit a patient to the ICU requires considering several clinical and nonclinical factors. Studies have investigated factors associated with the decision but have not explored the relative importance of different factors, nor the interaction between factors on decision-making. We examined how ICU consultants prioritize specific factors when deciding whether to admit a patient to ICU.

**Design:**

Informed by a literature review and data from observation and interviews with ICU clinicians, we designed a choice experiment. Senior intensive care doctors (consultants) were presented with pairs of patient profiles and asked to prioritize one of the patients in each task for admission to ICU. A multinomial logit and a latent class logit model was used for the data analyses.

**Setting:**

Online survey across U.K. intensive care.

**Subjects:**

Intensive care consultants working in NHS hospitals.

**Measurements and Main Results:**

Of the factors investigated, patient’s age had the largest impact at admission followed by the views of their family, and severity of their main comorbidity. Physiologic measures indicating severity of illness had less impact than the gestalt assessment by the ICU registrar. We identified four distinct decision-making patterns, defined by the relative importance given to different factors.

**Conclusions:**

ICU consultants vary in the importance they give to different factors in deciding who to prioritize for ICU admission. Transparency regarding which factors have been considered in the decision-making process could reduce variability and potential inequity for patients.

## Abstract 2

Supplemental Digital Content is available in the text.

The decision whether to admit a patient to the ICU can be complex and difficult. ICU provides potentially lifesaving treatments unavailable elsewhere in the hospital. For patients unlikely to survive this can mean enduring invasive and distressing therapies rather than benefiting from supportive ward-based or palliative care. Often doctors make such decisions in situations of clinical uncertainty, with limited time, and unable to discuss treatment with the patient due to the severity of their illness. There are few relevant prognostic indicator tools and limited professional guidance to support doctors making these decisions. It is therefore unsurprising that there is substantial variability in how such decisions are made (1).

Studies have explored a range of factors that may influence whether a patient is admitted to ICU including severity of acute illness (2–9); severity of comorbidities (5, 10–13); functional status of patient (3, 5, 8, 14–19); clinical trajectory of patient’s condition (13, 16, 20, 21); patient’s age (3, 5, 6, 11, 13, 14, 22, 23); patient’s gender (11, 23–25); insurance status of patient (in United States) (12) and availability of ICU resources (2–5, 7, 12, 13, 15, 17). These studies are of variable quality, and heterogeneity of results make it difficult to draw generalizable conclusions. Further, methods used do not allow for comparison of the relative importance (RI) of these factors, or exploration of interactions between factors.

We examined how senior intensive care doctors (consultants) prioritize factors when making decisions about whether to admit a patient to ICU. We also investigated how they differ in their preferences.

## MATERIALS AND METHODS

### Design

The study used an economics approach, choice experiment (CE), widely used in healthcare to understand preferences in decision-making (26, 27). We determined how consultants used patient-related information to make ICU admission decisions, specifically whether a factor played a significant role in their decision-making; the type of influence it had (i.e., increase/decrease the probability of admission); and which factors exert the greatest influence on decisions.

### Development of the CE

This study was part of the project: “Understanding and improving the decision-making process surrounding admission to the intensive care unit” which included a systematic review of factors influencing ICU admission and an ethnographic study of the decision-making process at six U.K. hospitals (28).

A planned interim analysis of data from the systematic review and the ethnographic study was used to identify factors to be included in the CE. The systematic review identified 88 studies investigating factors associated with decisions around admission to ICU. We analyzed data from observations of 15 ICU referrals and interviews with 20 ICU doctors from two NHS hospitals in our ethnographic study, at which point no additional new information with regard to factors influencing admission decisions (the specific objective of this analysis) was emerging from the data.

We coded observation field notes and interview transcripts for influences on the decision-making process and categorized codes into factors that were mapped to factors identified in the systematic review to check for congruence and any additional factors. For example, the gestalt assessment of the patient (the “look” of the patient) did not feature as a factor in the literature but emerged from the qualitative data. **Table S1** (Supplemental Digital Content 1, http://links.lww.com/CCM/E838) provides more detail of how the systematic review and qualitative data informed the development of the CE.

The final list of factors included in the CE (**Table 1**) were all patient-related. Factors were allocated levels corresponding to clinical situations observed during the ethnographic study. Severity of acute illness of the patient was included as both physiologic variables and the U.K. National Early Warning Score. Levels of comorbidities were selected to reflect comparable stages of disease: peridiagnosis, established disease, advanced disease with limited survival.

**TABLE 1.**
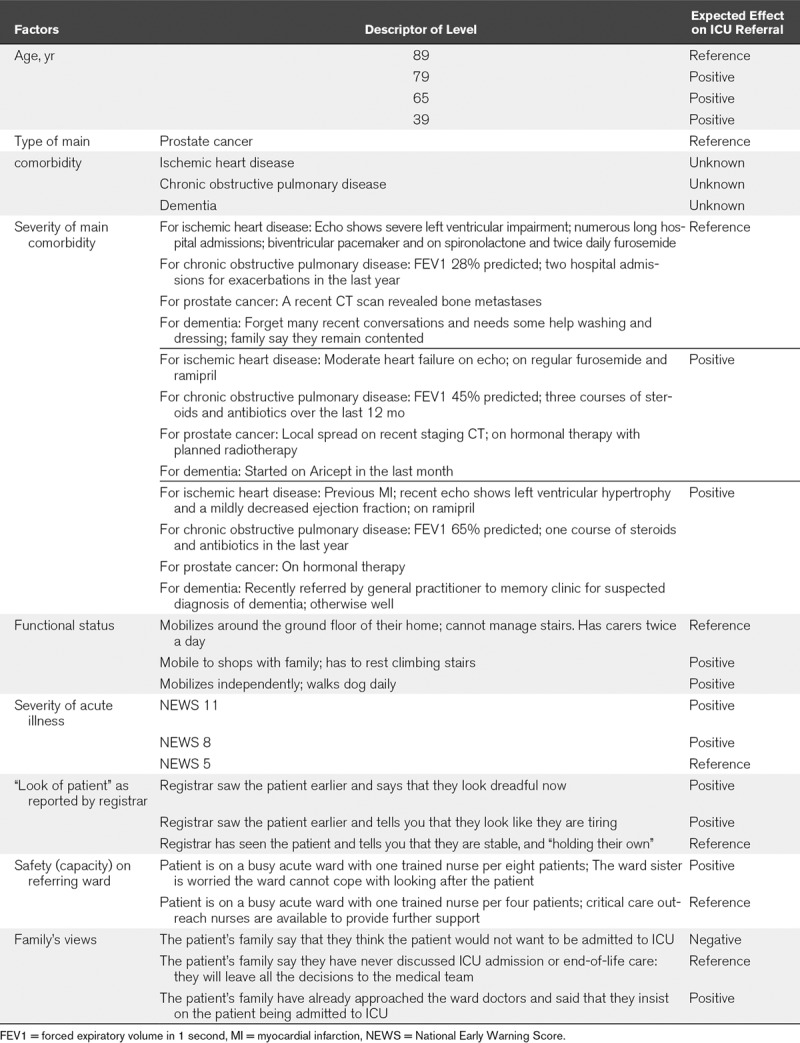
Factors and Levels in the Choice Experiment

**Figure 1.**
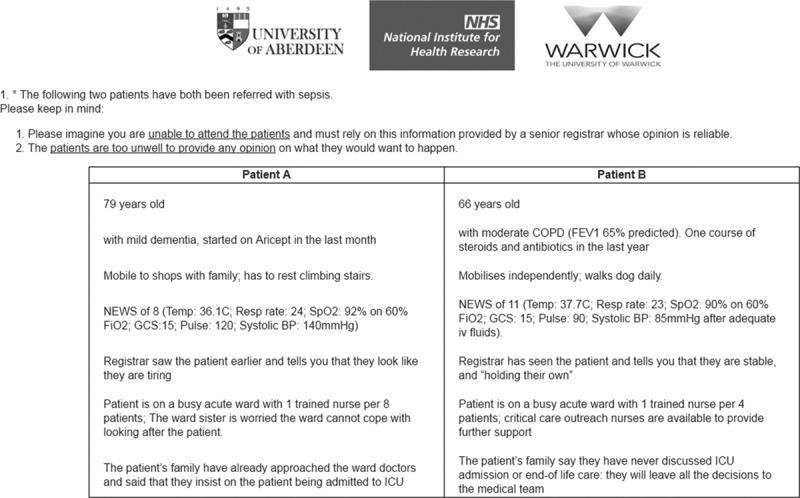
Illustration of the choice task format. BP = blood pressure, COPD = chronic obstructive pulmonary disease, FEV1 = forced expiratory volume in 1 second, GCS = Glasgow Coma Scale, NEWS = National Early Warning Score, NHS = National Health Service, Spo_2_ = pulse oximetry.

Patient profiles were generated using experimental design methods (29), resulting in 24 choice tasks. To reduce cognitive burden, each respondent faced 12 choice tasks. A warm-up choice task and two data quality check tasks were added. In each choice task, two hypothetical patient profiles were presented, and participants were asked three related questions: 1) would you admit patient A? (yes/no); 2) would you admit patient B? (yes/no); and 3) which patient should be given priority for admission? (patient A/B) (****Fig. 1****). Information was collected on participants’ sociodemographic characteristics and response times. The CE tool was delivered online by ClinVivo Limited http://www.clinvivo.com/.

### Participant Recruitment

We recruited NHS hospitals through regional clinical research networks (that support recruitment to research across the NHS). In participating hospitals, an invitation to participate and link to the CE was distributed to senior ICU doctors (consultants). An invitation was also emailed to all consultant members of the U.K. intensive care society. Participants could indicate which hospital they worked at, but this was not required. No financial incentive was received. Completion of the survey was interpreted as implied consent. Ethical approval for the project was obtained from the Coventry and Warwickshire Research Ethics Committee (15/WM/0025).

### Sample Size

Using standard sample size calculations for CEs, a minimum of 146 ICU consultants were required (30). We doubled this to explore how preferences differed among ICU consultants. See **supplementary material** (Supplemental Digital Content 1, http://links.lww.com/CCM/E838) for information on sample size calculations.

### Analysis

We assessed the quality of the choice data using standard criteria (desirability; stability; logical consistency; response time; see supplementary material, Supplemental Digital Content 1, http://links.lww.com/CCM/E838). We specified a multinomial logit (MNL) model to estimate the effects of changes in patient-related factors (e.g., increasing patient’s age from 66 to 79 yr) on the probability of admitting the patient to ICU. We report odds ratios, indicating changes in the likelihood of a patient’s admission to ICU when one factor changes. Using the MNL variable estimates, we calculate the RI each attribute makes to the referral decision; this is calculated as the difference in the range of attribute’s variable values. We calculate percentages from these relative ranges, obtaining a set of attribute importance values that add to 100% (31).

Differences among ICU consultants in their preference patterns for patient admission were estimated using a latent class logit model (32). We again estimate RI scores for attributes, as described above. Given that eight factors were used to describe patients’ profiles, a perfectly balanced decision-making would result in a 12.5% score of RI for each factor (100/8). This was used as a benchmark to determine whether the consultants’ decision-making is biased toward any factor. We analyzed effects of consultants’ personal characteristics on their membership of a preference pattern group.

We further explored preferences by investigating the relationship between type and severity of comorbidity, that is, does the importance of type of comorbidity in the referral decision depend on severity of comorbidity? To do this, we reestimated the MNL model with additional interaction effects between preferences for type and severity of main comorbidity.

## RESULTS

The CE opened in April and remained open until we had achieved the necessary sample size, closing in June 2016 with 303 consultants from at least 48 different U.K. hospitals, completing the questionnaire. (The Faculty of Intensive Care Medicine [FICM] database includes 2,377 consultants). Our sample reflects the gender and age mix of ICU consultants in the United Kingdom; 79.5% of respondents were male (compared with 78.2% of FICM registered consultants), 21.1% were under 40 years old and 28.1% over 50 years old. The 2017 FICM unpublished workforce survey (39% response rate) reported that 19% of consultant responders were under 40 and 37% over 50 years (Faculty of Intensive Care Medicine. 2017 Workforce Survey. personal communication, 2019). Most respondents (76.9%) had worked in the ICU for more than 10 years and 33.6% worked in a university hospital. All respondents will have completed ICU specialty training.

The quality of responses was high, with 73.6% of participants meeting all four quality criteria. No participants failed more than two tests. There was no systematic relationship between consultants’ personal characteristics and the quality of their choices (supplementary material, including **Tables S2** and **S3**, Supplemental Digital Content 1, http://links.lww.com/CCM/E838). All responses were included in the final analyses.

### Impact of Patient-Related Factors on Referral Decisions

All eight factors had a significant effect on the decision to admit (**Table 2**). All three age-related variables were significant and positive, with younger patients more likely to be admitted. Patients with good functional status, more severe acute illness, subjectively reported as struggling by the registrar, on a ward with reduced nursing capacity, or whose family insist on admissions were more likely to be admitted.

**TABLE 2.**
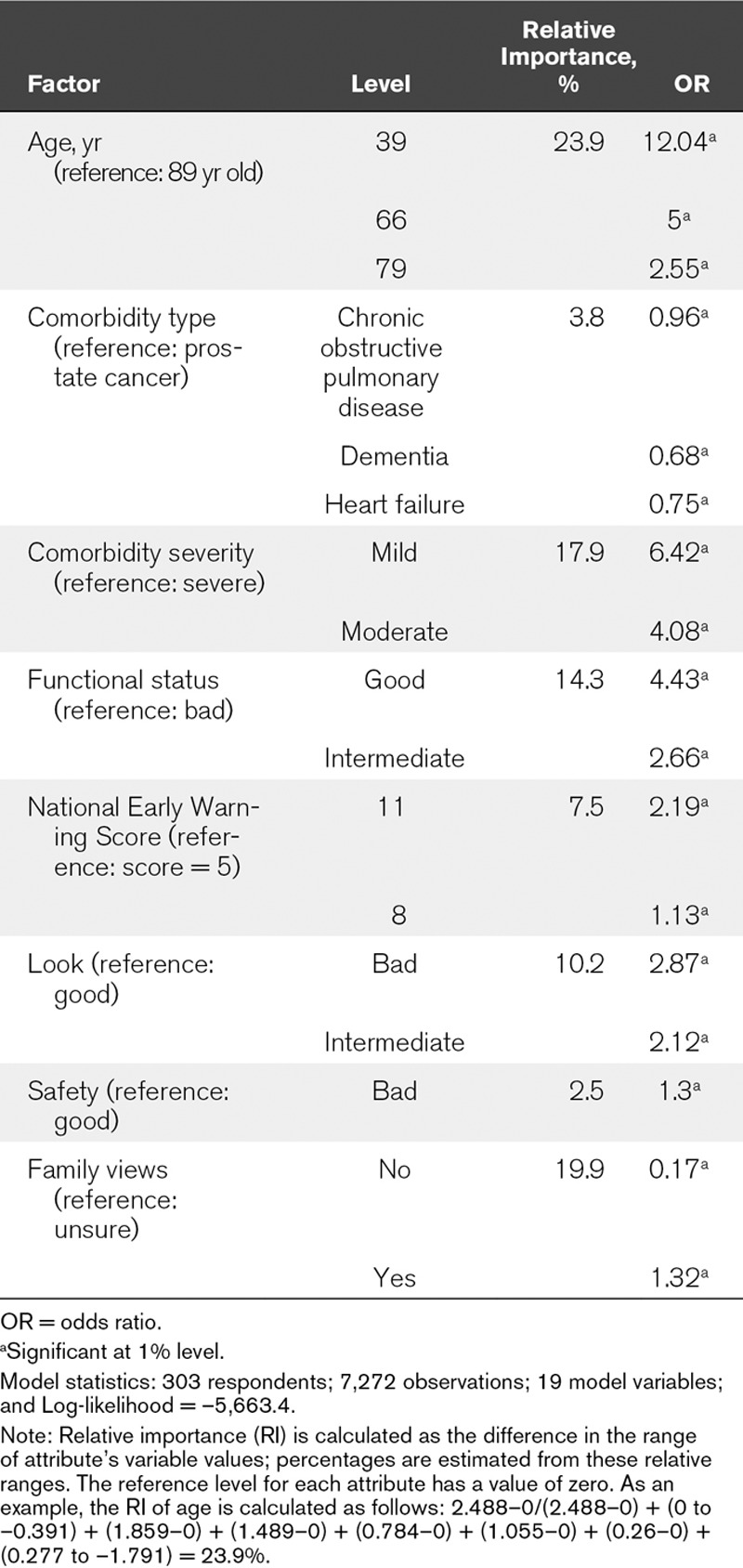
Impact of Patient-Related Factors on ICU Intensivists’ Admission Decisions

Patients’ age had the largest influence on consultants’ decisions (RI = 23.9%) with 39-year-old patients 12 times and 66-year-old patients five times more likely to be admitted than 89-year-old patients. This is followed by family views (RI = 19.9%). When the family is against admission, the patient is six times less likely to be admitted. The third most important effect is severity of comorbidity (RI = 17.9%). Patients with mild comorbidity are 6.4 times more likely to be admitted than those with severe comorbidity. Least important are type of main comorbidity (RI = 3.8%), patient’s safety in non-ICU ward (RI = 2.5%), and the severity of acute condition (RI = 7.5%). Patients with chronic obstructive pulmonary disease (COPD), heart failure, or dementia are 1.04, 1.34, and 1.48 times less likely to be admitted than patients with prostate cancer.

### Differences Among ICU Consultants in Their Preferences for Patient Admission

**Figure 2.**
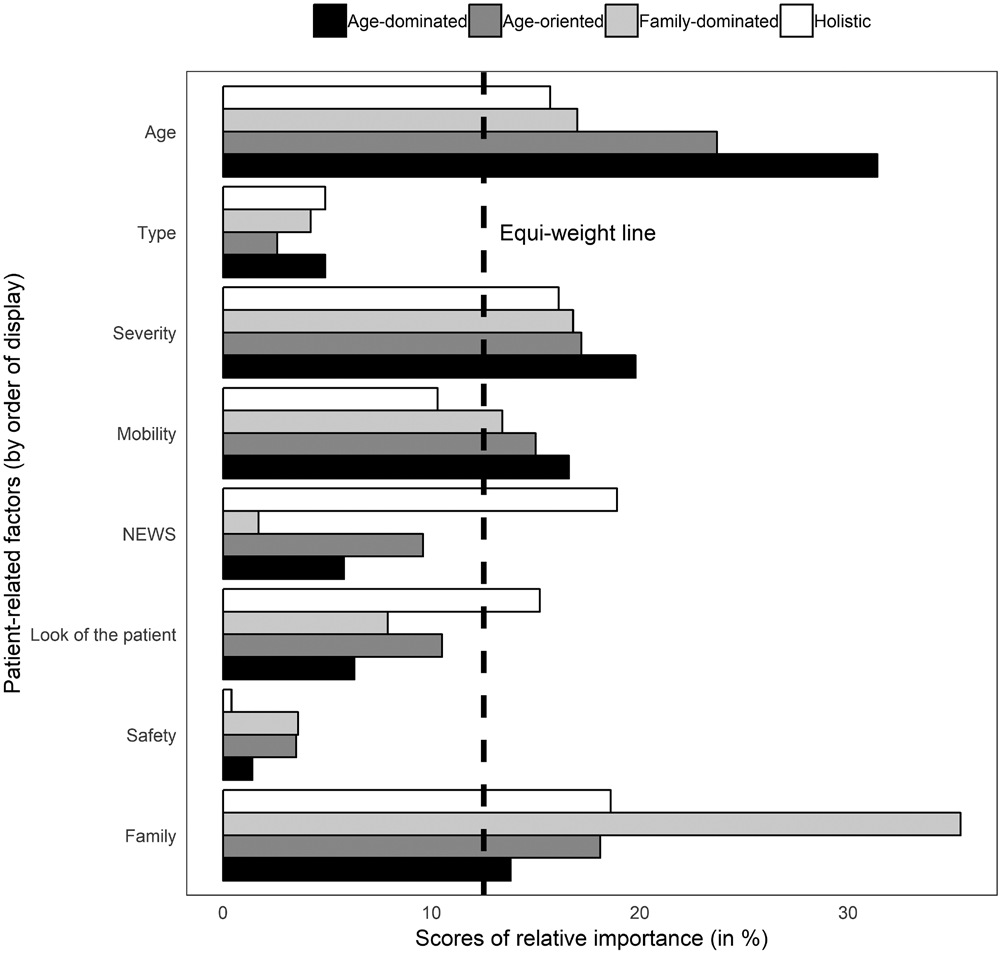
Comparison of relative importance scores across the four preference patterns identified among respondents. The *dashed line* indicates all attributes have equal importance, that is, relative importance = 12.5% (100/8). NEWS = National Early Warning Score.

Four preference patterns were identified (for detailed results, see ****Fig. 2**** and **Table S4**, Supplemental Digital Content 1, http://links.lww.com/CCM/E838). Preference pattern 1 is described as “age-oriented” (giving relatively more weight to age); 2 as “age-dominant” (decisions based mainly on age); 3 as “holistic” (similar importance to all factors); and 4 “family-dominant” (decisions mainly driven by family’s views). These four patterns represent 31% (pattern 1), 33.2% (pattern 2), 17.4% (pattern 3), and 18.4% (pattern 4) of participants.

### Effects of Consultants’ Personal Characteristics on Their Preference Patterns

Six effects reach significance at the 5% level (**Table S5**, Supplemental Digital Content 1, http://links.lww.com/CCM/E838): consultants older than 40 years are more likely to belong to preference pattern 1 and 3 than 4 compared with younger consultants. This is especially true for consultants older than 50 years. Consultants working in a medium-size ICU (11–19 beds) and in a University hospital are less likely to belong to preference patterns 1 and 3, respectively.

### Interaction Between Type and Severity of Comorbidity

**Figure 3.**
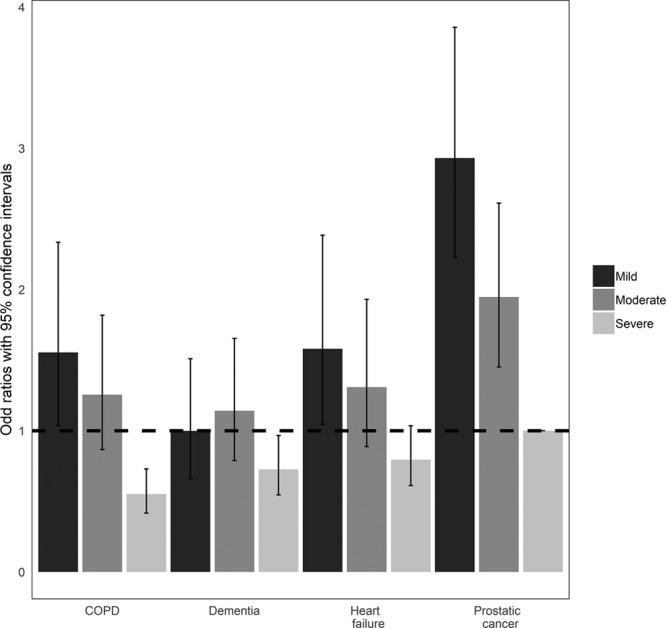
Associations between severity of comorbidities and likelihood of admission to ICU. The *dashed line* indicates a null effect on consultants’ admission decisions (i.e., odds ratio = 1) with severe prostate cancer as the reference category. All other effects are estimated relative to this reference category. Corresponding model estimates are in supplementary material (Supplemental Digital Content 1, http://links.lww.com/CCM/E838). COPD = chronic obstructive pulmonary disease.

Increasing severity of all comorbidities was associated with a decreased likelihood of admission to ICU; however, differences were observed across comorbidities (****Fig. 3****; for corresponding data, see **Table S6**, Supplemental Digital Content 1, http://links.lww.com/CCM/E838). For a mild level of severity, patients in all four comorbidity groups were more likely to be admitted than patients with severe prostate cancer. However, for moderate severity, the probability of ICU admission fell only in patients with COPD. At the most severe level, dementia was the comorbidity most likely to result in the patient not being admitted to ICU, followed by heart failure, then COPD.

## DISCUSSION

This study investigated the RI ICU consultants give to patient-related factors when deciding whether to admit to ICU. Of the factors examined, the most impactful are patient’s age, views of their family, and severity of main comorbidity. The acute physiologic variables of the patient had less impact than the subjective assessment of the registrar about how ill the patient looked. Both these acute illness assessments had less impact than age, comorbidity, and functional status. Four preference patterns emerged: “age oriented,” “age dominant,” “holistic,” and “family dominant.” Notably, the importance given to physiologic variables as an indicator of severity and to views of the patient’s family significantly differs across preference patterns. We also found that the relative effect of the type of comorbidity depends on the severity of that comorbidity.

Numerous studies have shown that increasing age is associated with refusal of admission to ICU (3, 5, 6, 11, 13, 14, 22, 23). Older patients often have several comorbidities and reduced physiologic reserve compared with younger patients. Our study suggests that age has an influence independent of this association. It may be that ICU consultants are consciously or subconsciously discriminating against older patients, or that there is an implicit linking of age with reduced capacity to benefit over and above other objective considerations. Alternatively, consultants may use age as a proxy for capacity to recover when other information such as functional reserve or comorbidity is unavailable, and this heuristic is maintained even when specific information is known. It is important that implicit assumptions are made explicit and justified to avoid unfair discrimination, particularly in the context of an aging population and equality legislation.

Existing literature supports our finding that severity of the patient’s acute illness is not the primary factor influencing admission decisions. Studies including multivariate analysis of severity of acute illness assessed by a variety of measures have shown no clear effect on decision-making (2–9) despite an association with patient outcomes (33). In our ethnographic study, ICU consultants expressed a reluctance to rely on physiologic variables, placing more weight on their gestalt assessment of the patient. This is consistent with our respondents who were influenced more by the registrar’s subjective report.

Few studies have explored the effect of patient or family preferences at admission to ICU; those which have report mixed findings (13, 16, 18). Our results suggest family views, when known, would have an influence on these decisions, particularly if this view is that the patient would not want to be admitted. This may reflect the legal framework in the United Kingdom which requires clinicians to consult those close to the patient if the patient lacks capacity, and take their views on the patient’s wishes into consideration. However, there are practical difficulties in engaging with patients and families at the time these decisions need to be made so often their views are unknown. The use of advance directives and emergency care treatment plans can provide valuable information for clinicians (34), but more work is needed to explore how patient preferences can have a meaningful influence on these decisions.

Our finding that patients with mild severity of comorbidity are more likely to be admitted suggests participants assess these patients as more likely to benefit from ICU. Evidence from a recent U.K. study on patient outcomes following ICU admission supports this assessment (35). This prioritization reflects the gatekeeping role of ICU consultants in the United Kingdom, that is, responsible for minimizing burden of ICU treatment while maximizing potential benefit from a limited resource. The finding that for a given level of severity of comorbidity patients with COPD, heart failure, and dementia are less likely to be admitted than those with prostate cancer may also be linked to clinicians’ perception of the patient’s ability to benefit from ICU. There is evidence that ICU clinicians are overly pessimistic in estimating outcomes for patients with COPD and heart failure (36, 37). In a resource-limited situation, this undue prognostic pessimism may influence a clinician to prioritize admission for a patient who does not have these comorbidities.

We find, unsurprisingly, variability in consultants’ preference patterns. Clinical judgments are often made in complex and uncertain situations, where clinicians may rely on heuristics and be influenced by “availability bias” (where own experience with a condition has more importance than objective weighing of the evidence (38, 39). Transparency regarding which factors have been considered in the decision-making process could reduce variability and potential inequity for patients. Understanding by clinicians of their own cognitive biases (40) and what influences them is a necessary part of improving practice. With this in mind, and looking forward, we have developed decision-making simulators which consultants can use to observe how their probability of admitting a given patient would be influenced by changes in the patient’s profile. Consultants can also see to which preference pattern group they are more likely to belong (available at https://warwick.ac.uk/fac/med/research/hscience/sssh/research/intensive/). Similar studies in different healthcare systems and further qualitative exploration of the decision-making process will help to explicate and make more transparent the wider contextual influences on these difficult and complex decisions.

This is the first study to use a CE to look at RI of patient-related factors for decisions to admit to intensive care and explore the interaction between different factors on decision-making. A strength of our study is the use of observational data to inform the CE. We identified factors not seen in the literature but which our observations indicated were important in clinical practice, for example, “look of the patient” and capacity of the ward to deliver care safely. Data quality was high, providing confidence in responses. Our results support previous findings of the importance of age but also confirmed our qualitative findings on the influence of gestalt assessment on these decisions. However, the study is limited by its design in that the cases do not take account of non-patient related factors and thus may not reflect the complex reality of these decisions. Although our sample reflected the U.K. ICU consultant population with regard to demographic characteristics, there may be other characteristics that affect its representativeness, for example, responders are more likely to think this is an important issue. This study focused on practice in the U.K. NHS. Future research could replicate our study in different countries to investigate the effects of social, professional, and regulatory differences on consultants’ admission decisions.

## CONCLUSIONS

ICU consultants place more priority on the age of a patient, the views of their family, and the severity of their comorbidity than physiologic prognostic scores when making admission decisions. However, consultants vary in their decision-making and how they prioritize these factors. Transparency regarding how factors are considered in the decision-making process could reduce variability and potential inequity for patients.

## ACKNOWLEDGMENTS

We would like to express our thanks to the ICU consultants who participated in the survey.

## Supplementary Material
