# Supplementary material for: U.K. Intensivists’ Preferences for Patient Admission to ICU: Evidence From a Choice Experiment
Source: Crit Care Med. 2019 Oct 11;47(11):1522–30. doi: 10.1097/CCM.0000000000003903 (PMC6798748; doi:10.1097/CCM.0000000000003903)
Supplement: Supplementary file 1 [file ccm-47-1522-s001.docx]

**Supplementary material**

**Table S1. Mapping of factors across systematic review and ethnographic data analysis to inform Choice experiment (CE) development.**

| **Factor category** | **Factor** | **Investigated in studies in SR (N)** | **Identified in ethnographic data analysis** | **Informed development of CE** |
| --- | --- | --- | --- | --- |
| **Patient-related factors** | Type of acute illness | 24 | x | Used as constant in CE (pneumonia) |
|  | Severity of acute illness | 45 | x | Defined in CE by physiological parameters and National Early Warning Score (NEWS) |
|  | Presence of chronic illness | 28 | x | Four commonly encountered progressive conditions were selected as co-morbidities for the CE to represent different disease processes and conditions with either observed and/or previously studied influence on likelihood of admission to intensive care. |
|  | Severity of chronic illness | 19 | x | For each selected condition levels of severity developed from standard disease specific scales |
|  | Functional status/quality of life measures | 39 | x | Levels developed from the descriptors of functional status observed as being elicited by intensive care clinical staff during the qualitative study (to provide plausibility and realism to scenarios). Broadly map to disease specific functional classifications. |
|  | Nutritional status | 2 |  | Not used (only 2 studies and no qualitative data) |
|  | Length of hospital stay | 7 |  | Not included in CE (to maintain a manageable number of factor combinations) |
|  | Trajectory of illness | 7 | x | Not included in CE (to maintain a manageable number of factor combinations) |
|  | Presence of DNACPR | 9 |  | Not included in CE (to maintain a manageable number of factor combinations) |
|  | Age | 53 | x | Included in CE. levels selected by research team to align with socially recognised stages of adult life which may influence decision-making (mid working age, latter end of-working life, established old age, advanced old-age). |
|  | Gender | 30 |  | Not included in CE (to maintain a manageable number of factor combinations) |
|  | Ethnicity | 9 |  | Not included in CE (to maintain a manageable number of factor combinations) |
|  | Patient preference | 18 | x | Combined with family preference in CE |
|  | Family preference | 14 | x | Combined with patient preference in CE |
|  | Health insurance status | 6 |  | Not included in CE (not relevant for UK NHS) |
| **Clinician-related factors** | Seniority of ICU clinician | 12 | x | CE only sent to consultant grade demographic data on years in ICU |
|  | Seniority of referring clinician | 4 | x | Not included in CE (to maintain a manageable number of factor combinations) |
|  | Demography of ICU clinician | 2 |  | Demographic questions in survey |
|  | Physician’s attitude/personal views/experience | 3 | x | Not possible to include in CE |
|  | Prognostic pessimism | 2 |  | Not possible to include in CE |
| **Organisational-related factors** | ICU bed availability | 39 | x | CE included a bed limitation constraint |
|  | Decision maker present | 2 |  | Not included (focus on patient related factors) |
|  | Specialty of patient | 9 |  | Not included (focus on patient related factors) |
|  | Time of day | 7 |  | Not included (focus on patient related factors) |
|  | Experience/expertise of ward team | 1 | X (safety on ward (number/ expertise/ competence of staff) | Included as number of available nurses per bed on referring ward. This was a strong theme in the qualitative data although not identified in the systematic review |
|  | Hospital characteristics | 5 |  | Not included (focus on patient related factors) |
|  | Avoid conflict/litigation | 2 |  | Not included (focus on patient related factors) |
|  | Other | 3 |  | Not included (focus on patient related factors) |
| **Additional factors identified in qualitative data** | Patient dignity |  | x | Not possible to include in CE |
|  | Subjective ‘look of the patient’ |  | x | Included in CE as report from registrar |
|  | Likelihood of survival of patient |  | x | Subjective assessment captured in registrars assessment for CE |
|  | Likely to benefit from ICU care |  | x | This is the judgment informed by factors being investigated in CE  Subjective assessment captured in registrars assessment for CE |
|  | Lack of available information |  | x | Not possible to include in CE (see limitations of study) |
|  | Personal connection with patient |  | x | Not possible to include in CE |

**Data quality -** We use four criteria to approximate data quality:

1. *Choice desirability*: Task #15 set all eight patient-related features to their best (i.e., most attractive in terms of ICU admission) level and worst level for the patient A and B respectively. The respondent failed the desirability test when patient A was not prioritised over B.
2. *Choice stability*: Task #14 was a repetition of task #1. The rankings of the different choice options (i.e., patient A, patient B, none of them) are compared between the two tasks. The respondent failed the stability test when none of the top and last ranked options were not repeated.
3. *Logical consistency*: If A (B) should be admitted but not B (A), then B (A) should not be prioritised over A (B). This condition was verified for each task and respondent. We then computed, for each respondent, the number of tasks in which the condition was satisfied. If this proportion was lower than 80%, the respondent failed the logical consistency test.
4. *Response time*: We recorded response times at the task level for each participant. We the identified “speedsters” i.e., participants who answered the choice questions ‘too quickly’ to make decisions that would accurately reflect their preferences for patient admission. For each choice task, we computed the first quintile of the response time distribution. Then for each participant and each task we determined whether the observed response time was below the quintile value or not. If this proportion of very quick choices was higher than 50%, the respondent failed the response time test.

A respondent’s performance on the four tests was summarised into a quality score, ranging from 0 (when the participant passes the four tests) to 4 (when the participants fails the four tests). We excluded respondents who provided ‘low quality data’, defined as a quality score >= 3).

The quality of the consultants’ decisions was relatively high (Table S1). No consultant failed more than two tests and only one failed two test. A higher proportion of consultants (26 %) failed only one test.

**Table S2. Data quality tests**

We did not perform a sensitivity analysis investigating the effects of removing participants who failed at least one quality test; such exclusion would be extreme, could not be motivated from a theoretical point of view (the analytical model allows for random error) and is likely to remove valid information.

We analysed how data quality varied across the consultants. Using a logistic regression model our dependent binary variable = 1 if the consultant failed at least one quality check and 0 otherwise. Our independent variables were the personal characteristics of consultants. No characteristic reached significance at the 5% level.

**Table S3. Effect of consultants’ personal characteristics on the quality of their responses**

|  |  | **MLE** | **SE** | **P** |
| --- | --- | --- | --- | --- |
| **1. Model parameters** | | | | |
| Constant |  | -2.504 | 1.084 | 0.021 |
| Gender (Ref: Male) | *Female* | 0.152 | 0.333 | 0.648 |
| Hospital (Ref: Not university.) | *University.* | 0.601 | 0.319 | 0.060 |
| Age (Ref: <= 40 years old) | *41-50 years old* | -0.149 | 0.407 | 0.714 |
|  | *> 50 years old* | 0.066 | 0.554 | 0.905 |
| Experience in ICU (Ref: <= 4 years) | *5-9 years* | 1.250 | 1.099 | 0.255 |
|  | *10-14 years* | 1.172 | 1.094 | 0.284 |
|  | *15-19 years* | 1.617 | 1.120 | 0.149 |
|  | *>= 20 years* | 1.568 | 1.147 | 0.172 |
| ICU size (Ref: <= 10 beds) | *11-19 beds* | -0.285 | 0.345 | 0.408 |
|  | *>= 20 beds* | 0.034 | 0.427 | 0.936 |
| **2. Model statistics** | | | | |
| # Individuals | | 303 | | |
| # Cases | | 80 | | |
| # Parameters | | 11 | | |
| Log-likelihood | | -169.1 | | |
| MLE: Maximum likelihood estimate; SE: Standard error; P: P-value | | | | |

**DCE sample size calculation**

Given we did not have a prior information about the preferences of consultants it was not possible to perform a formal sample size computation (power analysis). Following good practice, we based our sample size computation on a formula for choice proportions [31]:

$$N\geq\frac{1-P}{\mathrm{TP}\left( \beta^{2} \right)}\left[ \phi^{-1}\left( \frac{1+\alpha}{2} \right) \right]^{2}$$

where “T” is the number of choice tasks per participant; “J” the number of choice options per task; “β” accuracy level; “α” confidence level and “P” is the expected choice probability (when no prior information about individuals’ preferences is known this corresponds to the chance level: 1 / J), and “ϕ” is the normal distribution (CDF).

For our study: T = 12; J = 3; β = 0.9; α = 0.05, leading thus to a minimum requirement of 65 respondents. We anticipated that up to 10% of respondents might need to be excluded because of data quality issues (i.e., high rate of missing values on key variables or low quality of the choices) giving a minimum sample size requirement of 73 respondents. As the choice tasks were divided between two different versions of the questionnaire, this was doubled to 146. We double this requirement to enable us to explore preferences heterogeneity (2 x 146 = 292; 303 recruited).

**Table S4. Differences among ICU consultants in their preferences for patient admissions**

|  |  | **"Age-oriented"** | | **"Age-dominated"** | | **"Holistic"** | | **"Family-dominated"** | |
| --- | --- | --- | --- | --- | --- | --- | --- | --- | --- |
|  |  | **Odds ratio** | **Relative importance** | **Odds ratio** | **Relative importance** | **Odds ratio** | **Relative importance** | **Odds ratio** | **Relative importance** |
| **Age** (Reference: 89 years old) | *39 years* | 17.37 | 24% | 48.86 | 31% | 2.98 | 16% | 8.63 | 17% |
|  | *66 years* | 5.74 |  | 12.14 |  | 2.24 |  | 4.68 |  |
|  | *79 years* | 2.91 |  | 4.71 |  | 1.55 |  | 1.2 |  |
| **Co-morbidity type**  (Reference: Prostate cancer) | *COPD* | 1.16^a^ | 3% | 1.2 | 5% | 1.15 | 5% | 1.47 | 4% |
|  | *Dementia* | 1.21^a^ |  | 1.53^a^ |  | 1.18^a^ |  | 1.16^a^ |  |
|  | *Heart failure* | 1.36^a^ |  | 1.41^a^ |  | 1.22^a^ |  | 1.19 |  |
| **Co-morbidity severity** (Reference: Severe) | *Mild* | 7.94 | 17% | 11.7 | 20% | 3.08 | 16% | 7.96 | 17% |
|  | *Moderate* | 4.34 |  | 5.54 |  | 2.34 |  | 8.39 |  |
| **Functional status** (Reference: Bad) | *Good* | 6.08 | 15% | 7.78 | 17% | 1.82 | 10% | 5.48 | 13% |
|  | *Intermediate* | 3.17 |  | 3.58 |  | 2.05 |  | 2.85 |  |
| **NEWS** (Reference: score = 5) | *11* | 3.17 | 10% | 2.06 | 6% | 2.78 | 19% | 1.08^a^ | 2% |
|  | *8* | 1.26 |  | 1.55 |  | 1.35^a^ |  | 1.14 |  |
| **Look** (Reference: Good) | *Bad* | 3.54 | 10% | 2.19 | 6% | 2.89 | 15% | 2.73 | 8% |
|  | *Intermediate* | 2.55 |  | 1.45 |  | 2.83 |  | 1.55 |  |
| **Safety** (Reference: Good) | *Bad* | 1.52 | 3% | 1.19 | 1% | 1.03 | 0% | 1.57 | 4% |
| **Family views** (Reference: Unsure) | *No* | 7.88^a^ | 18% | 4.45^a^ | 14% | 3.02^a^ | 19% | 40.21^a^ | 35% |
|  | *Yes* | 1.12 |  | 1.24 |  | 1.21 |  | 2.2 |  |
| ^a^ These Odds Ratios have been reversed to indicate how likely the patient is to not be admitted. | | | | | | | | | |
| Model statistics: 303 respondents; 7,272 observations; 75 model parameters; Log-likelihood = -5,392.9. | | | | | | | | | |

**Table S5. Effects of consultants’ personal characteristics on their preference patterns**

|  |  | **"Age-oriented"** | **"Age-dominated"** | **"Balanced"** |
| --- | --- | --- | --- | --- |
|  |  | **(Reference: "Family-dominated")** | | |
|  |  | **Odds Ratio** | | |
| Constant | | 2.11 | 1.69 | 0.79 |
| Gender (Reference: Male) | *Female* | 0.77 | 1.1 | 1.68 |
| Hospital (Reference: Not univ.) | *Univ.* | 1.07 | 0.84 | 0.34^b^ |
| Age (Reference: Less than 40 years old) | *41-50 years old* | 3.71^b^ | 2.43 | 6.57^b^ |
|  | *> 50 years old* | 16.3^a^ | 4.9 ^c^ | 12.7^b^ |
| Experience in ICU (Reference: Less than 4 years) | *5-9 years* | 0.63 | 1 | 0.23 |
|  | *10-14 years* | 0.89 | 1.53 | 0.27 |
|  | *15-19 years* | 0.29 | 0.44 | 0.15 |
|  | *>= 20 years* | 0.19 | 0.24 | 0.17 |
| ICU size (Reference: Less than 10 beds) | *11-19 beds* | 0.33^b^ | 0.61 | 1.15 |
|  | *>= 20 beds* | 0.62 | 1.26 | 2.32 |
| Model statistics: Respondents = 303; Observations = 7,272; Log-likelihood = -5,368.7 | | | | |
| ^a^ Effect significant at 1% level; ^b^ Effect significant at 5% level; ^c^ Effect significant at 10% level | | | | |

**Table S6. Interaction between type and severity of co-morbidity**
